# The Oral–Gut Microbiome Connection in Patients with Periodontitis: A Systematic Review

**DOI:** 10.3390/medicina62061133

**Published:** 2026-06-10

**Authors:** Damaris Anton, Mihaela Băciuț, Oana Almășan

**Affiliations:** 1 Department of Prosthetic Dentistry and Dental Materials, “Iuliu Hațieganu” University of Medicine and Pharmacy, 400006 Cluj-Napoca, Romania; damaris.anton@elearn.umfcluj.ro (D.A.); oana.almasan@umfcluj.ro (O.A.); 2 Department of Oral and Maxillofacial Surgery and Implantology, Faculty of Dental Medicine, “Iuliu Hațieganu” University of Medicine and Pharmacy, 400012 Cluj-Napoca, Romania

**Keywords:** periodontitis, gut, microbiome, dysbiosis, inflammation

## Abstract

*Background and Objectives*: This study aims to evaluate the recent literature on the oral–gut connection in the context of periodontal disease, emphasizing the significance of systemic risk associated with chronic inflammation. This review explores whether chronic inflammation resulting from periodontal disease can induce systemic conditions through alterations in the gut microbiome and whether periodontal treatment may contribute to overall health improvement. *Materials and Methods*: A systematic database search was performed using pre-established search strategies. Searches were conducted in three databases between 1 and 20 October 2025. A total of 578 articles were screened for eligibility based on inclusion and exclusion criteria. Two authors agreed on the selection process used. The methodological quality of the included studies was assessed using the Newcastle–Ottawa scale and the Risk of Bias 2 Tool. *Results*: Eleven studies were considered eligible for inclusion in the review. The gut microbiome is similar to the oral microbiome in patients with periodontitis. Gut microbial shifts may drive systemic inflammation and metabolic dysfunction. Tooth loss and gum disease are linked to alterations in the gut bacteria, potentially compromising the intestinal barrier permeability. In contrast, the presence of natural teeth may prevent oral–gut bacterial transmission. Changes in the gut microbiota are correlated with improvements in periodontal status after non-surgical periodontal therapy. *Conclusions*: The evidence presented in this review supports an association between periodontitis, oral–gut microbial alterations, and systemic inflammatory conditions. However, most available studies are observational, limiting causal inference. Targeted modulation of the gut microbiome may represent a promising area for future research, but its clinical applicability remains inconclusive.

## 1. Introduction

Periodontitis is an infectious and host-mediated inflammatory disease associated with dysbiosis of subgingival bacterial biofilms, resulting in progressive alveolar bone loss and soft tissue destruction around teeth [1]. The prevalence of periodontitis in dentate adults between 2011 and 2020 was estimated to be around 62%, and 23.6% of the population had severe forms of periodontitis, indicating a higher prevalence than previous data from 1990 to 2010 [2,3,4].

Scientific data report many systemic implications of periodontal disease through toxins produced by periodontal pathogenic bacteria that can enter the bloodstream and cause endotoxemia, which is a source of chronic systemic inflammation that influences multiple diseases [5,6,7,8].

The oral cavity represents the second largest colony of microorganisms after the intestinal microbiota and serves as the primary gateway to the body [9]. The balance of the oral microbiome is indispensable for maintaining systemic health. Certain types of bacteria can spread from one location to another and are involved in systemic impairment [9]. Furthermore, oral bacteria can be swallowed and translocated to the upper and lower digestive tracts (esophagus, stomach, small intestine, and large intestine) [9]. Under certain circumstances, these bacteria can ectopically colonize the upper and lower digestive tracts, causing various digestive disorders, such as inflammatory bowel disease [10]. Bacterial species such as *Fusobacterium nucleatum*, *Porphyromonas gingivalis*, and *Klebsiella* spp. have been implicated in changes in the host response through molecular mechanisms, leading to intestinal impairment [9,10].

Periodontal pathogens can induce changes in the gut microbiota, barrier function, and immune system, increasing the risk of systemic diseases associated with low-grade inflammation. Ectopic colonization by periodontal pathogens can lead to intestinal microecological imbalance and inflammation, damaging the physical intestinal epithelial barrier [11,12]. Oral microbes have been detected in the lower gastrointestinal tract, suggesting their ability to survive gastric and bile acids [13]. The altered functional barrier of the intestine, manifested as increased intestinal permeability in intestinal dysbiosis, modifies the immune response and changes the profile of bacterial metabolic products [14]. The consequences are systemic inflammation, altered glucose and lipid metabolism, and worsening of pre-existing systemic conditions. The theory that the general status of the host influences the development and progression of periodontitis is supported; thus, dysbiotic intestinal microbiota could be involved in the onset and progression of periodontal disease [15].

Another important aspect observed in patients with periodontitis is the entry of bacterial lipopolysaccharides from the periodontal pockets into the bloodstream. This triggers a chronic inflammatory state in the body, predisposing individuals to multiple systemic diseases [16]. The primary source of endotoxemia is the intestinal microbiota, and in healthy patients, the intestinal barrier plays a crucial role in preventing the entry of bacterial lipopolysaccharides into the circulation. The mucin layer on the intestinal wall also plays a significant role in this process. In the context of intestinal dysbiosis, intestinal permeability increases, allowing the translocation of lipopolysaccharides into the blood, a phenomenon known as metabolic endotoxemia [16,17]. The translocation of oral pathogenic bacteria to the gut, initiating systemic propagation, is not merely a random event but is heavily facilitated by chronic inflammatory diseases (periodontitis) combined with compromising factors such as reduced gastric acid (PPI use), increasing age, or pre-existing gut inflammation [18,19]. Among the periodontal pathogens implicated in oral–gut microbial interactions, *Fusobacterium nucleatum* and *Porphyromonas gingivalis* have been most consistently associated with gastrointestinal dysbiosis and systemic inflammatory conditions. Additionally, Tannerella forsythia, Treponema denticola, Prevotella intermedia, and Aggregatibacter actinomycetemcomitans have also been linked to oral dysbiosis and may contribute to oral–gut microbial translocation [18]. The evidence suggests that these microorganisms may contribute to alterations in the gut microbial composition, disruption of the epithelial barrier, immune activation, and chronic low-grade inflammation, thus promoting gut dysbiosis and a systemic inflammatory response [16,17,18].

Periodontitis has been shown to induce colonic inflammation in mice through suppression of the GPR109A receptor, which interferes with epithelial barrier integrity [20]. Non-surgical periodontal therapy, which removes pathogenic dental biofilms, plays a crucial role in reducing serum inflammatory markers, such as IL-6 and C-reactive protein, and in decreasing plasma trimethylamine N-oxide levels in mice, a molecule linked to atherosclerosis. Moreover, periodontal treatment can improve gut dysbiosis and intestinal barrier affected by periodontitis in animal models, acting as a therapeutic intervention not only locally but also systemically by shifting the dysbiotic gut composition to a healthier state [21,22].

The primary aim of this review is to evaluate the recent literature on the oral–gut connection in the context of periodontal disease, emphasizing the significance of systemic risk associated with chronic inflammation. This review seeks to explore whether chronic inflammation resulting from periodontal disease can affect systemic conditions through alterations in the gut microbiota and whether periodontal treatment might contribute to overall health improvement. While several previous reviews have linked oral health to systemic implications via the oral–gut relationship, this systematic review aims to highlight the crucial role of periodontal disease in driving systemic inflammation and the risk of certain diseases influenced by gut dysbiosis.

## 2. Materials and Methods

The systematic review was conducted and reported in accordance with the PRISMA 2020 guidelines [23]; the protocol was not registered in a public database.

A systematic database search was conducted using pre-established search strategies with MeSH and other specific keywords. The searches were performed in three electronic databases, PubMed/MEDLINE, Scopus, and Web of Science, between 1 and 20 October 2025. Queries were adapted for each database, as detailed in Table 1, and data range filters were applied to display only records from 2020 to 2025.

All records were imported into the Rayyan platform [24] (https://www.rayyan.ai/, version 1.7), accessed on 6 November 2025, to facilitate duplicate removal and the screening process. Articles that remained after duplicate removal were assessed for eligibility based on specific inclusion and exclusion criteria, as shown in Table 2. The screening process, which involved reading titles and abstracts, was carried out by two authors. If the information was too vague or missing, the full text of the article was accessed, so that only the relevant articles were included in the review. In cases of disagreement over whether to include an article, a third author (MB) made the final decision.

All of the included articles were assessed by two authors (A.D. and O.A.) to determine the final decision for inclusion by reading the full-text version. The screening process concluded on 6 November 2025, with all authors in agreement.

The methodological quality of the included observational studies (cohort and case–control) was independently assessed by two reviewers using the Newcastle–Ottawa scale (NOS). This tool evaluated studies across three domains: the selection of study groups, the comparability of groups, and the ascertainment of exposure or outcome. Each study could receive a maximum of nine stars. Studies scoring 7–9 stars were considered high quality, 4–6 denoted moderate quality, and 3 or fewer indicated low quality. Disagreements between reviewers were resolved through discussion. The cross-sectional studies were assessed using the newly adapted version of NOS, structured in four domains with a maximum score of 16 points [25].

The risk of bias of the randomized clinical trial and the prospective interventional trial included in our review was assessed using the Cochrane Risk of Bias 2 (RoB 2) Tool, the revised version, and the ROBINS-I tool, provided through the platform https://www.riskofbias.info/welcome [26,27]. The RoB 2 assessed potential sources of bias arising from five domains: randomization process, deviations from intended interventions, missing outcome data, measurement of the outcomes, and selection of the reported results. The ROBINS-I tool for prospective interventional trials evaluated bias across seven domains related to confounding factors, selection of participants, intervention classification, deviations from intended interventions, missing data, outcome, and selective reporting. Two authors independently assessed the studies, and disagreements were discussed until they reached a consensus.

## 3. Results

**Figure 1:**
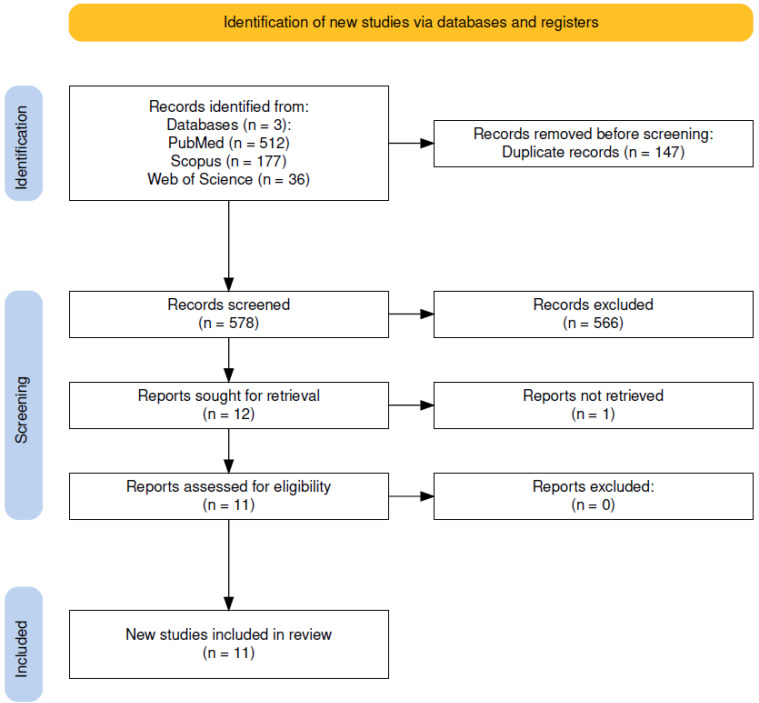
Prisma flow diagram [28].

A total of 725 records from the three electronic databases were imported into the Rayyan platform [24], and 147 records were removed as duplicates. After the duplicate removal, 578 records remained for the screening process, and 566 records were excluded. Twelve reports were considered eligible for full-text retrieval; however, one full-text article could not be obtained, resulting in 11 reports assessed for eligibility and ultimately 11 studies included in the review (Figure 1).

Two authors assessed the 11 full-text version articles to extract data, including title, authors, publication year, study type, subjects, periodontal definition, sample type, microbiome analysis, confounders, and main findings. The articles were categorized by key characteristics that link periodontitis to gut dysbiosis and systemic conditions (Table 3, Table 4 and Table 5). Two additional summary tables were created to improve understanding of the reviewed evidence. Table 6 organizes the biological levels of evidence supporting the oral–gut axis in periodontitis, while Table 7 provides a summary of the clinical interpretation of periodontal therapy and the associated oral–gut microbiome outcomes across the included interventional studies.

Considerable heterogeneity was observed in the periodontal disease definitions used across the included studies. While some study methodologies employed current AAP/EFP staging and grading systems, others defined periodontal status using broader clinical criteria, including tooth loss, poor oral health, gum disease, or periodontal pocket depth (PPD) measurements. This variability should be considered when comparing the microbiota findings across studies.

## 4. Discussion

The observational studies included in our review were assessed for methodological quality based on their design (cross-sectional, cohort, and case–control). All studies demonstrated moderate-to-good methodological quality as assessed by the Newcastle–Ottawa scale (NOS) (Table 8) and its adapted version for cross-sectional studies (Table 9). Case–control and cohort studies generally demonstrated stronger methodological rigour due to clearer case definition, longitudinal follow-up, and more solid exposure and outcome assessments. Conversely, cross-sectional studies exhibited methodological limitations, particularly concerning small sample sizes, lack of reported sample size calculations, and limited representativeness of study populations, as many were conducted in single clinical centres or pilot cohorts. In addition, several studies offered limited adjustment for potential confounding factors, which could affect the interpretation of associations between periodontal disease, oral–gut microbiota alterations, and systemic conditions. Despite these limitations, most studies applied validated clinical periodontal assessments and advanced microbiome sequencing techniques, enhancing the reliability of exposure and outcome measurements.

**Figure 2:**
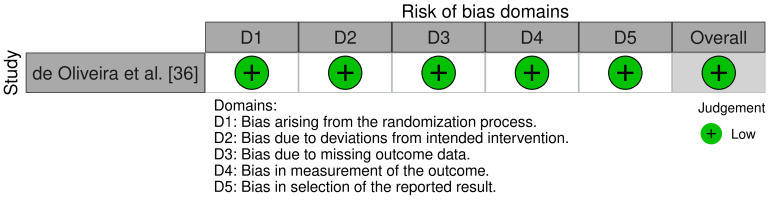
Cochrane risk of bias assessment results [40].

**Figure 3:**
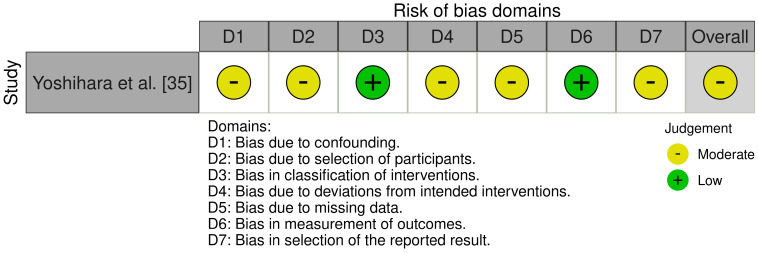
ROBINS-I assessment results [40].

The randomized clinical trial conducted by Oliveira et al. [36] was assessed using the RoB 2 tool (Figure 2) and was concluded to have a low risk of bias across all domains. This result indicates that the study used appropriate randomization methods, performed effective blinding, minimized the missing outcome data, and provided objective outcome measurements. The non-randomized interventional trial by Yoshihara et al. [35], evaluated with the ROBINS-I tool (Figure 3), exhibited a moderate risk of bias overall. This was mainly attributed to potential confounders, non-random participant selection, and the limited adjustments for factors that influence gut microbiota composition.

The evidence included in this review presents several strengths but also important limitations that should be considered when interpreting the main findings. A major strength found in the reviewed studies is the use of advanced molecular techniques, including high-throughput sequencing and metabolomic profiling, which allow detailed characterization of the oral and gut microbiota and provide relevant outcome measurements [41]. The inclusion of different study designs, such as observational and interventional studies, contributes to a broader understanding of the potential interactions between periodontal disease and the gut microbiota. Several limitations were identified across the included studies. Most studies followed an observational design, many of which were considered cross-sectional, which limits the ability to establish a causal relationship. Many studies had small sample sizes and did not provide a clear justification for sample size calculation. Another important limitation is the lack of adjustment for confounding variables that are known to influence gut microbiota composition, such as diet, antibiotic use, systemic diseases, and lifestyle factors [42,43,44]. Other observed aspects are the heterogeneity in study populations, the methods used for microbiome sampling, and analytical methods that may affect the comparability of results across studies. Consequently, while the current evidence suggests a consistent association between periodontitis and alterations in gut microbial composition, these findings should be interpreted with caution.

### 4.1. Direct Evidence Supporting the Oral–Gut Microbial Translocation

Among the various levels of evidence supporting the oral–gut axis in periodontitis, the strongest support for potential microbial translocation is provided by studies demonstrating the presence of oral-associated bacteria throughout the gastrointestinal tract, increased oral–gut microbiota similarity, and greater convergence between oral and gut microbiota profiles in disease states. This evidence can be classified into different levels of biological plausibility, ranging from indirect ecological associations to more direct evidence of microbial translocation, as summarized in Table 6.

Several studies reported increased similarity between the oral and gut microbiota in patients with systemic inflammatory conditions, suggesting a possible interaction between oral dysbiosis and gut microbial alterations. In patients with inflammatory bowel disease, including ulcerative colitis and Crohn’s disease, oral pathogenic taxa were enriched in the gut microbiota [29]. Similarly, poor oral health, characterized by tooth loss and periodontal disease, was associated with alterations in the composition and structure of the colonic mucosa-associated microbiota, potentially contributing to impaired intestinal integrity and function [30]. In contrast, elderly patients retaining a greater number of natural teeth demonstrated relatively stable gut microbiota profiles despite oral microbial composition resembling that of periodontitis, suggesting that natural dentition preservation may be associated with greater oral–gut stability [31].

Additional evidence supporting oral–gut microbial interactions comes from studies identifying oral-associated taxa in fecal samples under specific clinical conditions. In pregnant women with periodontitis, alterations in the subgingival microbiota were associated with distinct faecal microbial signatures, including enrichment of Coprococcus, which effectively discriminated between patients with periodontitis and those without [34]. Likewise, Yin et al. [45] reported increased faecal abundance of typically oral genera, including Porphyromonas, Streptococcus, Fusobacterium, and Veillonella, in women with preterm birth, accompanied by a reduction in beneficial gut commensals such as Coprococcus. While these findings support the existence of oral–gut microbial interactions, they primarily reflect ecological associations and compositional shifts in microbial communities rather than conclusive evidence of active bacterial translocation.

Some of the strongest mechanistic evidence supporting a potential oral–gut translocation pathway has been reported for *F. nucleatum* in colorectal cancer. Strain-level genomic sequencing demonstrated genetic similarity between *F. nucleatum* isolates identified in colorectal tumours and strains recovered from the saliva of the same patients, suggesting a possible relationship between the oral cavity and tumour microenvironment [35,46]. These findings provide stronger support for microbial transmission than studies based primarily on microbial diversity associations and suggest that certain oral pathogens may survive gastrointestinal transit and colonize intestinal tissues under disease conditions.

The potential clinical relevance of *F. nucleatum* translocation is further supported by studies linking this periodontal pathogen to gastrointestinal oncogenesis. In patients with metabolic dysfunction-associated steatohepatitis-related hepatocellular carcinoma (MASH-HCC), Fusobacterium abundance was significantly increased compared with patients without carcinoma, whereas *P. gingivalis* was associated with gut microbial alterations linked to gastrointestinal cancer risk [33]. Furthermore, a meta-analysis of 10 studies demonstrated that fecal *F. nucleatum* abundance has high sensitivity and specificity as a complementary biomarker for colorectal cancer diagnosis [47]. Notably, non-surgical periodontal therapy was associated with reduced faecal *F. nucleatum* levels in patients with colorectal tumours, further supporting the oral cavity as a potential source of intestinal translocation by this pathogenic bacterium [35].

Collectively, these findings offer stronger support for the existence of oral–gut microbial transmission pathways in periodontitis. Nevertheless, most available evidence is observational, and the precise mechanisms underlying bacterial survival, gastrointestinal translocation, and long-term persistence within the intestinal ecosystem are not yet fully understood.

### 4.2. Ecological Dysbiosis, Gut Microbiota Alterations and Associated Systemic Inflammatory Response

In addition to studies suggesting potential microbial translocation, a substantial portion of the current literature describes broader ecological alterations affecting both the oral and gut microbiota in periodontitis. These findings indicate microbial shifts associated with dysbiosis and changes in microbial abundance, rather than direct evidence of bacterial transmission between the oral and gastrointestinal tracts.

Several studies reported significant alterations in the gut microbial composition in patients with periodontitis, including reduced microbial diversity, enrichment of pro-inflammatory-associated taxa, and depletion of beneficial commensal microorganisms [31,32,39]. Such dysbiotic patterns were associated with systemic inflammatory and metabolic dysfunction, suggesting that oral dysbiosis may contribute to broader ecological alterations within the gut microbiota. Lin et al. [32] demonstrated that patients with periodontitis exhibited distinct gut microbial signatures characterized by increased pathogens and altered metabolic pathways associated with inflammation. Similarly, Miyauchi et al. [39] observed gut microbiota dysbiosis in periodontitis, accompanied by changes in intestinal immune homeostasis and systemic inflammatory response.

In the reviewed studies, the reverse interaction of the oral–gut axis was less extensively explored. Although several studies reported gut dysbiosis, depletion of beneficial commensals, and enrichment of pro-inflammatory microbial communities in association with periodontitis and systemic diseases, direct evidence demonstrating that specific intestinal bacteria contribute to periodontal inflammation remains limited [31,32,39]. Consequently, the currently available evidence mainly indicates a connection between changes in gut microbiota and periodontal disease, rather than a clear causal relationship.

### 4.3. Systemic Disease Associations and Clinical Implications

The potential clinical relevance of the oral–gut axis has gained increasing attention, particularly in relation to systemic diseases. Colorectal cancer has emerged as one of the most extensively investigated. *F. nucleatum* has been consistently identified in colorectal tumours and fecal samples, suggesting a potential role in inflammation, immune modulation and oncogenesis [33,35,46,47]. Similar associations have been described in patients with metabolic dysfunction-associated steatohepatitis-related hepatocellular carcinoma (MASH-HCC), where increased *F. nucleatum* and *P. gingivalis* associated with gut dysbiosis were linked to gastrointestinal cancer risk [33]. These findings support the possibility that oral pathogens associated with periodontitis may contribute to a pro-inflammatory intestinal state under specific disease conditions.

The oral–gut axis has been implicated in metabolic disorders such as type 2 diabetes mellitus, where chronic low-grade inflammation and altered gut microbial composition may contribute to insulin resistance and metabolic dysfunction [32]. The relationship between periodontitis and rheumatoid arthritis (RA) further illustrates the potential systemic implications of the oral–gut axis. Both conditions share overlapping inflammatory pathways, and alterations affecting the oral and gut microbiota have been associated with chronic immune activation and dysbiosis in patients with RA [37]. *F. nucleatum* was found enriched in the gut microbiota of patients with RA and has been shown to exacerbate arthritis in experimental models [48]. In addition, methotrexate therapy was associated with reduced alpha diversity in both the oral and gut microbiota and altered the relationships between microbial and clinical parameters of RA and periodontitis [37]. Recent evidence also reported enrichment of pathogenic taxa and depletion of beneficial commensals in both the oral and gut microbiota of female patients with RA, further supporting a possible relationship between periodontal dysbiosis and systemic inflammatory dysregulation [49,50].

Among the studies included in this review, *F. nucleatum* and *P. gingivalis* were the most frequently associated periodontal pathogens with gut microbial alterations and systemic disease conditions [33,35]. *F. nucleatum* was identified in association with colorectal cancer and demonstrated the strongest evidence supporting a potential oral–gut transmission pathway [35]. *P. gingivalis* was linked to gut microbial alterations associated with gastrointestinal cancer risk [33]. Other periodontal pathogens implicated in the oral–gut interactions, including Tannerella forsythia, Treponema denticola, Prevotella intermedia, and Aggregatibacter actinomycetemcomitans, were primarily discussed in supporting studies in the literature rather than directly investigated in the studies included in this review [18].

Collectively, these findings suggest that the oral–gut axis may represent a biologically and clinically relevant pathway linking periodontitis with systemic diseases characterized by chronic inflammation and microbial dysbiosis. However, the predominantly observational nature of the available evidence limits causal interpretation.

### 4.4. Clinical Interpretation of Periodontal Therapy and Microbiota Changes

The impact of non-surgical periodontal therapy (NSPT) on gut microbiota composition remains inconclusive, particularly in short-term studies characterized by heterogeneous treatment protocols and variable periodontal clinical responses. Several studies included in this review indicated that while NSPT may effectively modify the oral microbiota and reduce periodontal pathogenic burden, its effects on gut microbial dysbiosis are limited or inconsistent [35,36,37,38,39,51]. Notably, the objectives and intensity of periodontal therapy varied substantially across the included studies, depending on the associated systemic condition and severity of periodontal disease. In medically compromised patients, such as those with colorectal cancer or rheumatoid arthritis, periodontal therapy primarily aims to reduce oral inflammatory burden through conservative approaches, including scaling and oral hygiene improvement. In contrast, for patients with advanced periodontitis, therapeutic goals may also include periodontal stabilization, preservation of periodontal attachment, and surgical treatment of deep lesions [52,53]. As summarized in Table 7, interpretation of microbiota alterations following periodontal therapy requires consideration of the periodontal clinical outcomes reported in each study. It is important to recognize that the performance of periodontal therapy should not be equated with the immediate resolution of periodontal inflammation. Clinical periodontal improvement may vary considerably depending on baseline disease severity, systemic condition, smoking habits, oral hygiene practices, maintenance care, and patient compliance. Therefore, interpretation of oral–gut microbiota alterations following NSPT requires careful consideration of periodontal clinical parameters, including probing depth, bleeding on probing, clinical attachment level, and plaque control.

Overall, the current evidence does not demonstrate that non-surgical periodontal therapy consistently restores gut eubiosis. While some studies reported reductions in specific taxa, such as fecal *F. nucleatum*, and partial shifts towards healthier microbial states, others demonstrated persistent gut dysbiosis despite improvements in periodontal clinical outcomes [35,36,37,38,39]. These findings suggest that periodontal therapy may influence certain aspects of oral–gut microbial interactions, but its effects on restoring gut microbial homeostasis remain uncertain.

The use of probiotics as an adjunct to periodontal therapy is considered a biologically plausible and generally safe strategy for modulating microbial communities along the oral–gut axis. However, current evidence is insufficient to support their routine clinical application for improving oral–gut dysbiosis in patients with periodontitis, as reflected in current Clinical Practice Guidelines for periodontitis management [52,53]. Although some studies have reported that probiotic supplementation may improve periodontal clinical parameters and partially restore microbial balance by reducing pathogenic taxa and supporting beneficial commensals [54,55], the overall literature remains inconclusive regarding their ability to consistently reverse gut dysbiosis associated with periodontitis.

A relevant and insightful consideration relates to Helicobacter pylori, which has been investigated in relation to oral colonization, gastric infection, and gastrointestinal inflammation. The oral cavity represents a potential reservoir for bacterial persistence and reinfection. Previous studies have suggested associations between H. pylori, oral dysbiosis, and periodontal disease, although the nature and clinical significance of these relationships remain debated [56]. Furthermore, H. pylori has been shown to induce alterations in the gut microbiota and was linked to intestinal inflammation and systemic immune dysregulation [56]. However, none of the studies included in this review specifically evaluated the role of H. pylori regarding the oral–gut axis in periodontitis. Therefore, the available evidence from this review does not allow conclusions related to its contribution to the oral–gut microbial interactions.

### 4.5. Limitations and Methodological Heterogeneity

The findings presented in this review should be interpreted in light of substantial methodological heterogeneity among studies. Several included studies were characterized by relatively small sample sizes, which may limit the broadness and statistical reliability of the reported findings. Significant variability was observed in periodontal disease definitions and severity classifications, study populations, ethnicity, systemic comorbidities, smoking status, oral hygiene practices, and adjustment for confounding factors. Additionally, differences in microbiome methodologies, including sample type, sequencing approaches (16S rRNA sequencing versus shotgun metagenomics), taxonomic resolution, and analytical strategies based on microbial diversity or taxon abundance, may substantially influence the interpretation and comparability of microbiota findings across studies. Furthermore, most available evidence is observational and cross-sectional, which does not establish the directionality of the oral–gut relationship. It remains uncertain whether periodontitis contributes to gut dysbiosis, whether gut dysbiosis influences periodontal inflammation, or whether both conditions are influenced by shared behavioural, dietary, systemic, microbial, or inflammatory factors. Therefore, although current evidence supports a potential relationship between periodontitis, gut dysbiosis, and systemic inflammation, further longitudinal and mechanistic studies using standardized periodontal and microbiome methodologies are necessary to clarify the biological and clinical significance of these associations.

The authors acknowledge additional limitations. First, the review protocol was not previously registered, which may have reduced transparency regarding predefined eligibility criteria and outcomes. Second, restricted inclusion to open-access articles may have introduced selection bias by excluding potentially relevant studies available only through subscription-based access. Furthermore, the search strategy relied on specific keywords related to the gut microbiota and may not have identified all relevant studies using an alternative terminology, such as ‘intestinal microbiota’, ‘fecal microbiota’, ‘oral–gut axis’, ‘periodontal pathogens’, or specific bacterial taxa. Finally, although animal studies were intentionally excluded to maintain the clinical relevance of this review, mechanistic evidence regarding oral–gut microbial translocation often originates from experimental models. Therefore, the mechanistic interpretations presented in this review are based primarily on the included human studies and are supplemented by supporting studies in the literature.

## 5. Conclusions

The evidence reviewed suggests that periodontitis may have effects beyond the oral cavity and is associated with changes in the gut microbiota through a complex oral–gut axis influenced by microbial translocation, chronic inflammation, and interactions between the host and the microbial ecosystem. Oral dysbiosis may be linked to changes in the gut microbial composition and inflammatory pathways; however, the available evidence remains mainly observational and heterogeneous. Consequently, causality, directionality, and the clinical significance of these associations remain uncertain. While non-surgical periodontal therapy consistently improves oral microbial profiles and clinical periodontal parameters, its ability to restore gut dysbiosis remains limited. In addition, targeted modulation of the gut microbiota represents a promising area for future research, although current evidence is insufficient to support specific gut-targeted therapeutic interventions in patients with periodontitis.

## Figures and Tables

**Table 1:** Search strategies for each database.

Database	Query
PubMed	((“gastrointestinal microbiome”[All Fields] OR “intestinal permeability”[All Fields] OR “bacterial overgrowth”[All Fields] OR “intestinal barrier”[All Fields] OR “gut microbiome”[All Fields] OR “small intestinal bacteria overgrowth”[All Fields] OR “SIBO”[All Fields] OR “leaky gut”[All Fields] OR “zonuline”[All Fields] OR “endotoxemia”[All Fields]) AND (“periodontitis”[All Fields] OR “periodontal disease”[All Fields] OR “oral dysbiosis”[All Fields] OR “oral microbiome”[All Fields])) AND (y_5[Filter])
Scopus	TITLE-ABS-KEY((periodontitis) AND (microbiota) AND (gastrointestinal microbiome)) AND PUBYEAR > 2019 AND PUBYEAR < 2026
Web of Science	((TS = (periodontitis)) AND TS = (microbiota)) AND TS = (gastrointestinal microbiome)

**Table 2:** Inclusion and exclusion criteria.

Inclusion Criteria	Exclusion Criteria
Relevant topic	Reviews, systematic reviews and meta-analysis
Publication year 2020–2025	Animal model studies
Studies involving patients with periodontitis	Unavailable full text
Open-access articles	Irrelevant topic
English language	

**Table 3:** The gut microbiome reflects the oral microbiome in disease.

Title/Reference	Year of Publication/Author	Study Design/Subjects	Periodontal Definition	Sample Type	Microbiome Analysis	Adjusting Confounders	Main Findings
A potential pathogenic association between periodontal disease and Crohn’s disease [29]	2021 Jin Imai et al.	Prospective cohort study 69 patients with IBD and 50 healthy controls	Incipient periodontitis (the presence of 1 or more periodontal pockets deeper than 4 mm)	Saliva and stool	16S rRNA gene sequencing	Not clearly specified	In both ulcerative colitis (UC) and Crohn’s disease (CD) patients, the gut microbiome was more similar to the oral microbiome compared to healthy controls.
Oral Health and the Altered Colonic Mucosa-associated Gut Microbiota [30]	2022 Anthony A. Xu et al.	Cross-sectional observational study 63 participants	Poor oral health, tooth loss, and gum disease	Snap-frozen colonic biopsies	16S rRNA gene sequencing	Age, ethnicity, hypertension, diabetes, BMI, smoking, and alcohol use	There was a clear link between oral health status and the gut microbiota, suggesting that poor oral health could be a marker for systemic inflammation influenced by gut bacterial imbalances. Poor oral health, specifically tooth loss and gum disease, was linked to alterations in the community composition and structure of the adherent gut bacteria in the colon.
Integrated analysis of the oral and intestinal microbiome and metabolome of elderly people with more than 26 original teeth: a pilot study [31]	2023 Yuichiro Nishimoto et al.	Pilot observational study 10 elderly subjects with more than 26 natural teeth, 22 healthy controls and 24 subjects with periodontitis	Periodontitis and more than 26 natural teeth	Saliva and stool	16S rRNA gene sequencing	Not clearly specified	Elderly individuals with many natural teeth exhibited distinct oral microbiome and metabolome profiles that were similar to those of periodontal disease patients, despite their good oral health. Their gut microbiome and metabolome remained stable compared to healthy groups.

**Table 4:** The oral–gut axis mediates risk in multiple systemic conditions.

Title/Reference	Year of Publication/Author	Study Design/Subjects	Periodontal Definition	Sample Type	Microbiome Analysis	Adjusting Confounders	Main Findings
Periodontitis in elderly patients with type 2 diabetes mellitus: impact on gut microbiota and systemic inflammation [32]	2020 Jinyou Li et al.	Observational study 49 patients with T2DM (28 with periodontitis and 21 without periodontitis) and 29 age- and sex-matched healthy controls	Periodontitis (chronic periodontitis, aggressive periodontitis, gingivitis, periodontal abscess, periodontic-endodontic lesions)	Blood and stool	16S rRNA gene sequencing	Age- and sex- matched controls	Altered gut microbiota in patients with T2DM was linked to periodontal status and correlated with risk factors for bone loss and severity of disease. Circulating levels of pro-inflammatory mediators were higher in patients with T2DM and periodontitis than non-periodontitis T2DM. Gut microbial shifts caused by periodontitis influence systemic inflammation which may contribute to an unfavourable progression of T2DM.
Relationship of Metabolic Dysfunction-Associated Steatohepatitis-Related Hepatocellular Carcinoma with Oral and Intestinal Microbiota: A Cross-Sectional Pilot Study [33]	2024 Takaaki Matsui et al.	Cross-sectional pilot study 41 patients with metabolic dysfunction-associated steatohepatitis (MASH) and 19 patients with MASH–hepatocellular carcinoma (MASH-HCC)	Periodontitis	Saliva, stool and blood	16S rRNA gene sequencing	Not clearly specified	MASH-HCC affects periodontal pathogenic and intestinal bacteria. Fusobacterium occupancy is higher in MASH-HCC than in MASH patients. *P. gingivalis* affects intestinal bacteria associated with gastrointestinal cancer; thus, patients with MASH may require periodontal therapy to prevent gut dysbiosis.
Integrative microbiome and metabolome profiles reveal the impacts of periodontitis via oral-gut axis in first-trimester pregnant women [34]	2024 Tianfan Cheng et al.	Observational study 54 pregnant Chinese women (31 with periodontitis and 23 without periodontitis)	Maternal periodontitis following AAP/EFP classification	Subgingival plaque, saliva, serum and stool	16S rRNA gene sequencing	Gestational age, demographic characteristics, dietary habits, and sex hormone profiles	Periodontitis affects fecal microbiota and metabolites in pregnant women; as a novel bacterium, Coprococcus was identified in periodontitis patients.

**Table 5:** The impact of periodontal therapy on gut dysbiosis is inconclusive.

Title/Reference	Year of Publication/Author	Study Design/Subjects	Periodontal Definition	Sample Type	Microbiome Analysis	Adjusting Confounders	Main Findings
A prospective interventional trial on the effect of periodontal treatment on *Fusobacterium nucleatum* abundance in patients with colorectal tumours [35]	2021 Tsutomu Yoshihara et al.	Prospective interventional trial 31 patients with colorectal cancer included in the study	Periodontitis (based on JSP Clinical Practice Guideline for the Periodontal Treatment 2015) Severity was based on PPD values	Saliva and stool	16S rRNA gene sequencing and diversity analysis; digital PCR for *F. nucleatum* DNA levels	Not clearly specified	Patients who underwent successful periodontal treatment showed a significant decrease in fecal *F. nucleatum* levels. This reduction was not observed in patients whose treatment was unsuccessful. However, the treatment did not alter *F. nucleatum* levels in saliva or change the overall gut microbiota composition.
Impact of systemic probiotics as adjuncts to subgingival instrumentation on the oral-gut microbiota associated to periodontitis: a randomized controlled clinical trial [36]	2022 Adriana Miranda de Oliveira et al.	Randomized clinical trial 48 subjects with periodontitis	Untreated periodontitis with more than/or one site with PD ≥ 6 mm and more than/or two sites with PD ≥ 5 mm in different teeth	Subgingival biofilm and stool	16S rRNA gene sequencing	Randomized design	Systemic administration of a multi-species probiotic, as an adjunct to subgingival instrumentation, did not lead to an increase in gut microbial diversity or provide additional short-term clinical benefits in the treatment of periodontitis.
Methotrexate and Non-Surgical Periodontal Treatment Change the Oral-Gut Microbiota in Rheumatoid Arthritis: A Prospective Cohort Study [37]	2023 Sicília Rezende Oliveira et al.	Prospective cohort study 37 patients (27 with periodontitis) were evaluated at T0 32 patients (24 with periodontitis) at T1 28 patients (17 with periodontitis) at T2	Periodontitis (mild, moderate, severe)	Subgingival plaque and stool	16S rRNA gene sequencing	Not clearly specified	Both methotrexate and non-surgical periodontal treatment significantly impact the oral and gut microbiota in patients with rheumatoid arthritis. Methotrexate alters microbial diversity and correlations while non-surgical periodontal treatment changes oral microbiota.
Correlation in the change of gut microbiota with clinical periodontal parameters in grade C periodontitis patients after non-surgical periodontal therapy [38]	2025 Elif Mutafcilar Velioglu et al.	Pilot observational study 5 subjects with stage III, grade C periodontitis and 5 healthy controls	Stage III, grade C periodontitis	Saliva and stool	16S rRNA gene sequencing and qPCR reactions	Not clearly specified	Non-surgical periodontal therapy leads to clinical improvements and a beneficial shift in the gut microbiome in patients with periodontitis stage III, grade C, with a correlation between gut microbiota and periodontal probing depth and clinical attachment level.
Patients with periodontitis exhibit persistent dysbiosis of the gut microbiota and distinct serum metabolome [39]	2025 Eiji Miyauchi et al.	Cross-sectional observational study 23 patients with periodontitis stage III, grade B and 23 healthy controls	Stage III, grade B periodontitis	Saliva, serum and stool	16S rRNA gene sequencing	Not clearly specified	Periodontitis patients showed gut microbiota dysbiosis with decreased short-chain fatty acid producers. Periodontal therapy improved salivary microbiota but not gut microbiota.

**Table 6:** Biological evidence supporting the oral–gut axis in periodontitis.

Evidence Category	Main Findings	Representative Studies	Biological Interpretation	Main Limitations
Shared bacterial taxa between oral cavity and gut	Detection of oral-associated genera (*Fusobacterium*, *Porphyromonas*, *Streptococcus*, *Veillonella*) in fecal or intestinal samples	[34]	Suggests a potential oral contribution to gut microbial composition	Presence of shared taxa does not directly confirm active translocation or stable colonization
Increased oral–gut microbial similarity and gut dysbiosis	Altered microbial diversity, abundance shifts, depletion of beneficial commensals, and enrichment of pro-inflammatory taxa	[29,30,31,32,39]	Reflects ecological interactions between oral dysbiosis and intestinal microbial homeostasis	Diversity-based analyses cannot establish causality or microbial transmission pathways
Inflammatory and immune-related alterations	Intestinal barrier dysfunction, altered immune signalling, and chronic low-grade inflammation	[30,39]	Suggests possible mechanistic pathways linking oral dysbiosis with systemic inflammatory responses	Multifactorial inflammatory pathways and substantial confounding factors
Strain-level microbial similarity	Genomic similarity between oral and intestinal isolates of *Fusobacterium nucleatum*	[35]	Provides stronger support for potential oral–gut microbial transmission	Limited number of studies and mainly restricted to specific pathogens
Associations with systemic diseases	Associations between oral–gut dysbiosis and colorectal cancer, rheumatoid arthritis, diabetes mellitus, pregnancy complications, and MASH-HCC	[33,35,37]	Suggests possible clinical relevance of the oral–gut axis in systemic disease progression	Predominantly observational evidence, causality remains uncertain

**Table 7:** Clinical interpretation of periodontal therapy and oral–gut microbiome outcomes.

Study	Systemic Condition	Periodontal Intervention	Reported Periodontal Clinical Outcomes	Oral/Gut Microbiome Findings	Systemic/Inflammatory Implications
Yoshihara et al. [35]	Colorectal cancer	NSPT/SRP	Reduction in periodontal inflammation and improvement in periodontal parameters after treatment in the improvement group	Reduced fecal *F. nucleatum* abundance without major overall gut microbiota changes	Suggests the oral cavity as a potential source of intestinal *F. nucleatum* colonization
de Oliveira et al. [36]	-	NSPT/quadrant-wise SI (subgingival instrumentation) and systemic probiotics	Improvement in periodontal parameters in both groups	No significant improvement in gut alpha diversity or gut composition	Limited impact on gut dysbiosis despite oral clinical improvement
Oliveira et al. [37]	Rheumatoid arthritis	NSPT/full-mouth SRP and methotrexate therapy	Improvement in periodontal parameters after NSPT; MTX had no effect on periodontal parameters	Altered oral and gut alpha diversity and persistent gut dysbiosis	Suggests complex interaction between RA, treatment, and microbiome modulation
Velioglu et al. [38]	-	NSPT/full-mouth subgingival SRP	Reduction in probing depth and improvement in clinical attachment level correlated with a change in gut microbiota	Gut microbiome shifted toward healthy controls without statistical significance	Possible association between periodontal improvement and gut microbial changes
Miyauchi et al. [39]	-	Initial periodontal therapy, NSPT/SRP, and periodontal surgery for residual pockets	Periodontal treatment performed; all periodontal parameters significantly improved after treatment	Persistent gut dysbiosis despite periodontal therapy	Suggests that gut microbiome alterations may persist after short-term periodontal therapy

**Table 8:** Newcastle–Ottawa scale for quality assessment of cohort and case–control studies.

Reference	Study Design (Cohort/ Case–Control/ Cross-Sectional)	Adequate Case Definition	Representativeness of the Exposed Cohort/Controls	Selection of the Non-Exposed Cohort/Controls	Ascertainment of Exposure/ Definition of Controls	Demonstration That the Outcome of Interest Was Not Present at the Start of the Study	Comparability of Cohorts/Case and Controls Based on the Design or Analysis	Assessment of Outcome/Exposure	Was Follow-Up Long Enough for Outcomes to Occur?/Same Method of Ascertainment for Cases and Controls	Adequacy of Follow-Up of Cohorts/Non-Response Rate
Imai et al. [29]	Case–Control	*	*	*	*	-	-	*	*	-
Li et al. [32]	Case–control	*	*	*	*	-	**	*	*	-
Oliveira et al. [37]	Cohort	-	*	*	*	*	*	*	*	*
Velioglu et al. [38]	Cohort	-	*	-	*	*	*	*	*	*

**Note:** * indicates one star awarded; ** indicates two stars awarded; - indicates that no star was awarded.

**Table 9:** Newcastle–Ottawa scale quality assessment of cross-sectional studies.

Reference	Clarity of Stated Aim (0–2)	Sample Representativeness (0–2)	Sample Size (0–2)	Non-Respondents (0–2)	Exposure Assessment (0–2)	Control of Confounding Factors (0–1)	Comparability of Participants (0–1)	Assessment of the Outcome (0–2)	Statistical Tests (0–2)
Xu et al. [30]	2	1	0	0	2	1	1	2	2
Nishimo-to et al. [31]	2	1	0	0	2	1	0	2	2
Matsui et al. [32]	2	1	0	0	2	1	1	2	2
Cheng et al. [34]	2	1	0	0	2	1	1	2	2
Miyauchi et al. [39]	2	1	0	0	2	1	1	2	2

## Data Availability

The original contributions presented in this study are included in the article/Supplementary Materials. Further inquiries can be directed to the corresponding author.

## Supplementary Materials

The following supporting information can be downloaded at: https://www.mdpi.com/article/10.3390/medicina62061133/s1, Supplementary PRISMA 2020 Checklist [57].

## Abbreviations

The following abbreviations are used in this manuscript:

PPI	Proton pump inhibitors
IL-6	Interleukin-6
Spp.	Species
MeSH	Medical Subject Headings
NOS	Newcastle–Ottawa Scale
RoB 2	Cochrane Risk of Bias 2 Tool
ROBINS-I	Risk of Bias in Non-Randomized Studies of Interventions
UC	Ulcerative Colitis
T2DM	Type 2 Diabetes Mellitus
MASH	Metabolic Dysfunction-Associated Steatohepatitis
MASH-HCC	Metabolic Dysfunction-Associated Steatohepatitis-Related Hepatocellular Carcinoma
*P. gingivalis*	*Porphyromonas gingivalis*
*F. nucleatum*	*Fusobacterium nucleatum*
NSPT	Non-Surgical Periodontal Therapy
RA	Rheumatoid Arthritis
